# Transylvania University

**Published:** 1847-05

**Authors:** 


### TRANSYLVANIA UNIVERSITY.

By the Catalogue of the Medical Department of Transylvania University, which we have just received, we learn that the number of students in attendance there during the last session was 205. Of these, it is stated in the catalogue, nine were M.D.'s, and twenty took only the chemical ticket. The number of graduates was sixty-eight, two of whom had previously taken the degree at other institutions. The honorary degree was conferred upon Dr. Samuel B. Field, of Kentucky; Dr. Wm. H. Wilson, of Illinois; Dr. Wm. T. Le Grand, of Mississippi; and Dr. D. M. Lipscomb, of the same State.

The Lexington Medical Society has offered a prize of fifty dollars or a gold medal, for the best thesis submitted for the degree of M.D., in Transylvania University, at the next session. The same Society offers a similar prize for the best account of Continued Fever, as it prevails in any of the States out of New England.

